# Influence of ionic liquid and ionic salt on protein against the reactive species generated using dielectric barrier discharge plasma

**DOI:** 10.1038/srep17781

**Published:** 2015-12-10

**Authors:** Pankaj Attri, Thapanut Sarinont, Minsup Kim, Takaaki Amano, Kazunori Koga, Art E. Cho, Eun Ha Choi, Masaharu Shiratani

**Affiliations:** 1 Plasma Bioscience Research Center/Department of Electrical and Biological Physics, Kwangwoon University, Seoul 01897, Korea; 2 Graduate School of Information Science and Electrical Engineering, Kyushu University, Fukuoka, Japan; 3 Department of Bioinformatics, Korea University, Sejong 02841, Korea

## Abstract

The presence of salts in biological solution can affect the activity of the reactive species (RS) generated by plasma, and so they can also have an influence on the plasma-induced sterilization. In this work, we assess the influence that diethylammonium dihydrogen phosphate (DEAP), an ionic liquid (IL), and sodium chloride (NaCl), an ionic salt (IS), have on the structural changes in hemoglobin (Hb) in the presence of RS generated using dielectric barrier discharge (DBD) plasma in the presence of various gases [O_2_, N_2_, Ar, He, NO (10%) + N_2_ and Air]. We carry out fluorescence spectroscopy to verify the generation of ^•^OH with or without the presence of DEAP IL and IS, and we use electron spin resonance (ESR) to check the generation of H^•^ and ^•^OH. In addition, we verified the structural changes in the Hb structure after treatment with DBD in presence and absence of IL and IS. We then assessed the structural stability of the Hb in the presence of IL and IS by using molecular dynamic (MD) simulations. Our results indicate that the IL has a strong effect on the conservation of the Hb structure relative to that of IS against RS generated by plasma.

Cold temperature plasma (CAP) at atmospheric pressure provides a wide range of cutting-edge applications for various fields^1^,^2^,^3^,^4^,^5^,^6^. Researchers in plasma medicine have used different types of plasma devices among them atmospheric pressure plasma jet (APPJ) and dielectric barrier discharge (DBD) as the most common. These and other plasmas sources provide different applications for nanotechnology, chemical analysis, nanofabrication, material synthesis and plasma sterilization^7^,^8^,^9^,^10^. In recent years, there has been a sudden increase in the utility of plasma in the field of medicine^2^,^5^,^11^,^12^,^13^,^14^,^15^,^16^. Plasma medicine has been mainly dependent upon the specific action of various reactive species (RS) that affect various cellular functions. CAP contains a variety of UV, reactive oxygen species (ROS), charged particles, reactive nitrogen species (RNS), electrons, etc^17^,^18^,^19^,^20^,^21^,^22^. ROS and RNS, combined or independently, are well known to initiate different signaling pathways in cells and to promote oxidative stress^11^,^12^,^13^,^14^,^15^,^23^. Many researchers have performed experiments to identify the various RS in bio-solution and to determine their primary mechanism. CAP can generate RS that have selective action with living tissue, such as killing and healing activity^24^. In many areas, CAP has been proven to be useful, such as for wound healing, hospital hygiene, skin disease, sterilization, dental care, antifungal activity and cosmetics^25^,^26^,^27^,^28^,^29^. Recently, the potential of applying CAP in cancer therapy was also explored^2^,^11^,^12^,^13^,^14^,^15^,^23^. CAP treatment has also led to the selective eradication of cancer cells *in vitro* and a reduction of the tumor size *in vivo*^30^,^31^. ROS and RNS are known to be able to induce cell proliferation as well as cell death, and extreme amounts of RS can damage proteins, lipids, DNA, senescence and can induce apoptosis^5^,^11^,^12^. In addition, the extent of inactivation of microorganisms by the plasma depends upon the composition of the physiological fluids^32^,^33^. Nevertheless, the action of RS generated by plasma in the presence of various salts remains poorly understood.

Ionic liquids (ILs) are organic salts that are wholly composed of ions. The desired ILs can be designed with an appropriate choice of cationic and anionic components.^22^,^34^,^35^ The physicochemical properties of ILs include a high chemical/thermal stability, negligible vapor pressure, low toxicity, controllable hydrophobicity/hydrophilicity and conductivity^22^,^34^,^35^,^36^. This shows that such materials are promising as green alternatives to replace conventional volatile organic solvents (VOC). ILs can be used in numerous applications, including organic synthesis, electrochemical studies, extraction/separation, solar cells, and proteins^22^,^34^,^37^,^38^,^39^,^40^. Utility of biocompatible ILs have been greatly improved due to its by implementing handling and storage activities towards the biomolecules as compared to conventional aqueous medium.

Ionic salts (IS) have also been extensively used in food products and are a part of most animal fluids (including blood, sweat, and tears). IS are used in many physiological fluids to preserve microorganisms and enzymes^32^,^33^.

In this study, the protective action that the solute had on biomolecules during treatment with DBD plasma is assessed in the presence of different gases (O_2_, N_2_, Ar, He, NO (10%) + N_2_ and Air). We studied the structural changes in the hemoglobin (Hb) protein after the plasma treatment by using circular dichroism (CD) and fluorescence, assessed the generation of ^•^OH using fluorescence spectroscopy, and checked the generation of the H^•^ and ^•^OH by using the electron spin resonance (ESR) technique, all in the presence and absence of 2% (w/v) diethylammonium dihydrogen phosphate (DEAP) IL and 2% (w/v) sodium chloride (NaCl) ionic salt (IS). In addition, we also assess the structural stability of Hb in the presence and absence of DEAP IL and NaCl IS using molecular dynamics (MD) simulations.

## Results

In our previous study, we demonstrated that an ionic solution (IS) attenuates the toxicity of plasma on microorganisms during treatment^33^. This is probably due to the changes that IS has on the dynamics of the reactive species generated during plasma treatment. However, we observed in another study that IL can protect the structure and activity of enzymes against plasma toxicity^22^. Additionally, in our previous studies we observed that DEAP IL acts as the stabilizer to different proteins/cyclic dipeptides^40^,^41^,^42^. Therefore, the present study compares the action of DEAP IL and NaCl IS on the hemoglobin (Hb) structure during plasma treatment.

### Analysis of the radicals generated in the DBD plasma in the presence of different gases and study of the physical and chemical changes in water after treatment

**Figure 1:**
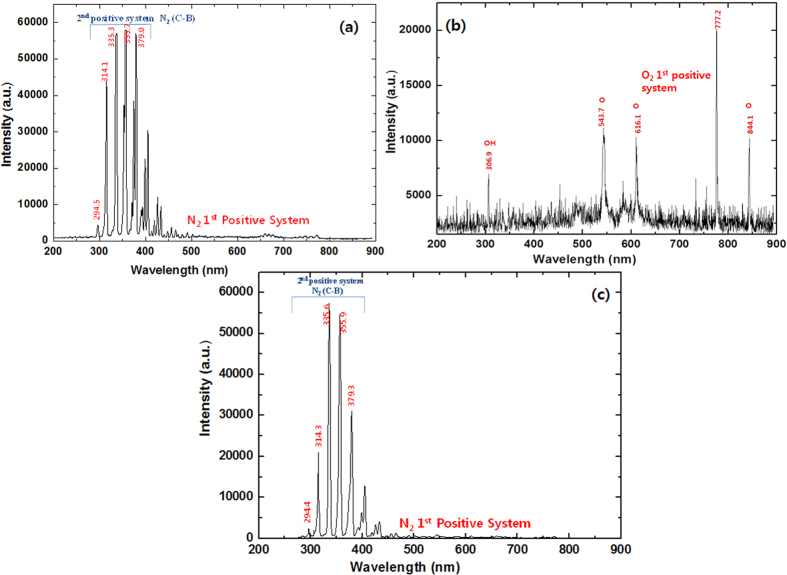
Optical emission spectra of (**a**) N_2_; (**b**) O_2_ and (**c**) NO (10%) + N_2_.

**Figure 2:**
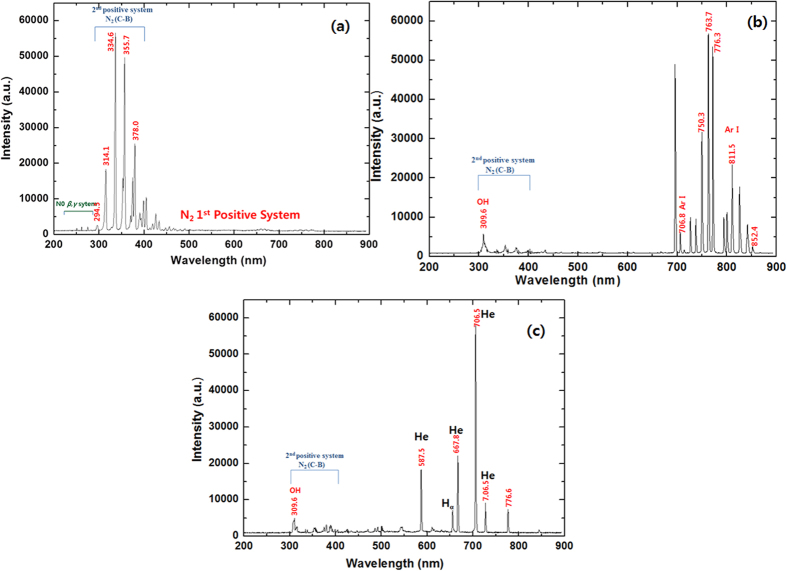
Optical emission spectra of (**a**) Air; (**b**) Ar and (**c**) He.

**Figure 3:**
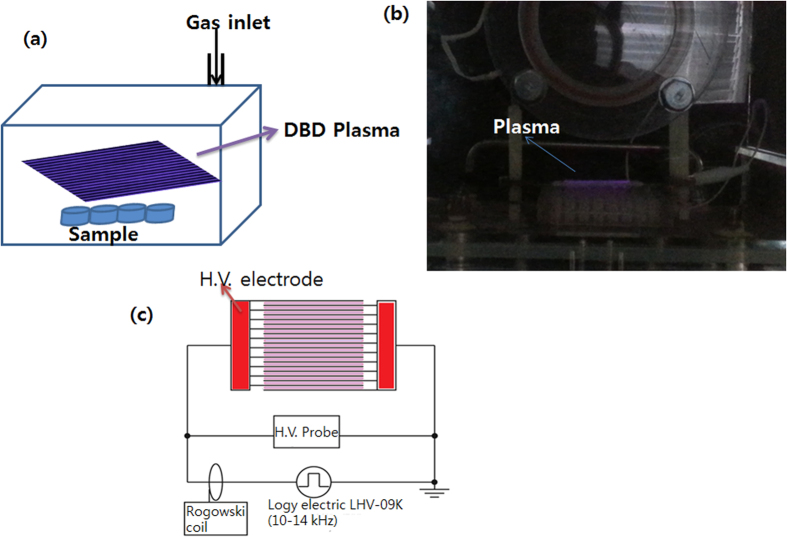
(**a**) Schematic diagram of the real treatment DBD treatment; (**b**) Actual treatment setup; (**c**) Schematic of the setup for DBD plasma.

The RS generated in the DBD plasma in the presence of different gases is detected via optical emission spectroscopy (OES), as shown in Figs 1 and 2. In the present work, we used O_2_, N_2_, Ar, He, NO (10%) + N_2_ and Air gases with DBD to generate plasma, as shown in Fig. 3. When we used the N_2_ feeding gas, we observed an N_2_ second-positive system (C^3^П_u_–B^3^П_g_) and an N_2_ first positive system (B^3^П_g_–A^3^П_u_^+^). For the N_2_ 2^nd^ positive system, we observed peaks at 294.5, 314.1, 335.3, 353.7 and 379.0 nm, but the N_2_ 1^st^ positive system lies between 500–700 nm, as shown in Fig. 1a. When O_2_ feeding gas was used in the DBD chamber, we observed a high-intensity ^•^OH peak at 306.9 nm, nascent oxygen atoms at 543.7 and 616.1 nm, and O_2_ 1^st^ positive system peaks at 777.2 and 844.1 nm, as shown in Fig. 1b. In contrast, we observed the N_2_ 2^nd^ positive system for NO (10%) + N_2_ at 294.5, 314.1, 335.3, 353.7 and 379.0 nm, which is the same as that for the N_2_ gas plasma spectra but the intensity of the peaks at 294.5, 314.3 and 379.3 are quite low for the NO (10%) + N_2_ gas plasma when compared to those for N_2_ gas plasma, as shown in Fig. 1c. Air plasma has small emission lines from a molecular NO β, γ system between 200 and 250 nm, but strong peaks where observed for the N_2_ 2^nd^ positive and weak peaks for N_2_ 1^st^ positive system, as illustrated in Fig. 2a. Moreover, for noble gas Ar and He plasma, we observed a small ^•^OH peak at 309.6 nm. We also found Ar and He lines between 600 and 800 nm, as shown in Fig. 2b,c.

Many of the radicals and electrons that are generated during plasma formation are either short lived or are long lived, but these radials or electrons reach a solution and form many complex reactions. These complex reactions result in the formation of other short- and long-lived radicals or species, and some of these long-lived species contribute to a change in the pH. The combined action of the electrons and radicals can affect the temperature of the solution, and hence, we have checked the pH and temperature of the solution with or without 2% (w/v) DEAP IL and 2% (w/v) IS after 10 min of DBD treatment in the presence of all gases, as shown in Figure S1. We observed that in DI water, the pH of the solution varies from gas to gas. The pH decreases the most for Air plasma and the least for Ar plasma. The pH also decreases the least for DEAP IL followed by IS and water. This trend in the different salts remains the same for all feeding gases, as shown in Figure S1a. We also observed that the change in pH for O_2_, N_2_ and NO (10%) + N_2_ was not too different. However, the change in the temperature was not much for the different gases in the DBD discharge for 10 min, and the presence of DEAP IL and NaCl IS does not have an influence on the temperature, as shown in Figure S1b. The temperature increases from 25 °C to 31 °C after a 10 min treatment, and this shows that during treatment, the temperature does not play a significant role in the deformation of the Hb structure. To understand the role that the stable species has in the pH decrease, we carried out ion chromatography for nitrogen species such as NO_2_^−^ and NO_3_^−^, as shown in Figure S1c,d. Our results show that both the NO_2_^−^ and NO_3_^−^ species are the highest in air plasma and the least in Ar plasma. Since the air plasma contains both oxygen and nitrogen species, these react together to form NO_2_^−^ and NO_3_^−^ during plasma treatment. Traces of nitrogen and oxygen that are dissolved in water might be present in the treatment chamber, which leads to the detection of the NO_2_^−^ and NO_3_^−^ species for other gases, even for noble gases.


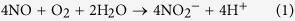



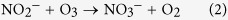


### Fluorescence and Electron paramagnetic resonance (EPR) analysis for ^•^OH detection in different gases plasma

In radical chemistry and in plasma chemistry, ^•^OH plays an important role because it is the neutral form of the hydroxide ion (OH^−^). Although there are many pathways to generate ^•^OH, four main pathways are the 1) decomposition of O_3_, 2) decomposition of nitrous acid, 3) decomposition of H_2_O_2_ and 4) reaction between the peroxyl radical (^•^HO_2_) with NO^43^. Several methods have been reported to detect ^•^OH, including optical absorption spectroscopy, fluorescence, high performance liquid chromatography (HPLC) and electron paramagnetic resonance (EPR)^43^.

**Figure 4:**
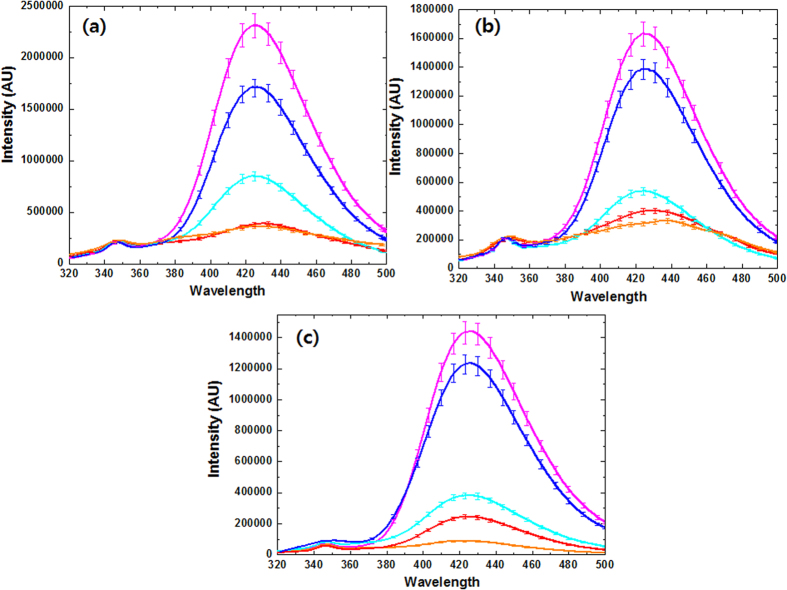
Fluorescence analysis of OH radicals using TA solution in (**a**) water; (**b**) 2% (w/v) NaCl solution and (**c**) 2% (w/v) DEAP IL solution after DBD plasma treatment with different feeding gases, such as Air (red), Ar (blue), He (cyan), NO (10%) +N_2_ (orange) and N_2_ (magenta).

^•^OH generation by DBD plasma in the presence of different feeding gases in water was monitored according to their reaction with the terephthalic acid (TA) anion to produce fluorescent hydroxy terephthalate (HTA) ions^44^,^45^,^46^. The electrons generated due to plasma collide with water molecules that generate hydroxyl radicals (^•^OH) and hydrogen radicals (H^•^). To investigate the generation of ^•^OH, we used terephthalate anions obtained by mixing terephthalic acid (TA) in alkaline aqueous solution. These terephthalate anions react with ^•^OH to create highly fluorescent hydroxyterephthalate ions (HTA)^44^, and this reaction is a very sensitive measure of the presence of ^•^OH, so many other research and groups have used this method to determine the generation of ^•^OH during plasma treatment^44^,^45^. We also used this method in our study to show how the formation of ^•^OH was also related to the gases used in the plasma chamber. This study provides a new way in which to use different gases in different experiments to test plasma medicines. We have used the several gases (O_2_, N_2_, Ar, He, NO (10%) + N_2_ and Air) in a chamber where we the DBD plasma is generated, and hence, during treatment, the main gas (environment gas) is the same as the treatment gas, therefore the interference of air is limited during treatment. We can also check that the conversion of the TA to HTA occurred through a fluorescence analysis. We have provided an excitation at ~310 nm and the emission was observed at ~425 nm for the formation of HTA. We found that during the fluorescence scan, there are two peaks with one at ~350 nm and other at ~425 nm. The small peak at ~350 nm is the unreacted or side product of TA during the reaction with ^•^OH to form HTA, while the peak at ~425 nm belongs to the formation of HTA formation, as shown in Fig. 4. The intensity of the fluorescence is directly related to the amount of HTA formation. First, we have studied the formation of HTA in water (DI) in the presence of N_2_, Ar, He, NO (10%) + N_2_ and Air gases plasma. We found that HTA is formed the most with the use of N_2_ gas as the feeding gas and the least with NO (10%) + N_2_ as the feeding gas.

Similar trends were observed in the presence of NaCl IS and DEAP IL, as shown in Fig. 4. However, the difference in the HTA formation using NO gas and Air gas plasma is not much for water, but the difference looks to be more clear for the IS and DEAP IL solution. To obtain a clear understanding of the amount of HTA formed from different gases, we have separately arranged the O_2_ gas plasma with other gases because the fluorescence intensity is very high for the O_2_ gas plasma, as shown in Fig. S2. In Figure S2, no peak is observed at ~350 nm for the O_2_ gas plasma, which is present in the other gas plasmas. This shows that the rate of conversion of TA to HTA is the maximum for the O_2_ gas plasma. Therefore, we can finally say that that the generation of ^•^OH depends upon the gas to gas interaction. Gases play a significant role in the formation of ^•^OH, and the final order to generate ^•^OH in our experiment was O_2_ > N_2_ > Ar > He > Air > NO (10%) + N_2_. Moreover, the intensity of the fluorescence related to the formation of HTA is less for NaCl IS and decreases further for the DEAP IL solution in all gas plasmas.

ESR spectroscopy is widely used due to its sensitivity and selectivity, and spin-trapping ESR can be used to detect various unstable radicals with a short life of even 1 ns^43^,^47^,^48^. In this work, we have used the spin-trapping EPR technique to detect H^•^ and ^•^OH generated during plasma treatment in the presence of NaCl IS and DEAP IL. We used 5, 5-dimethyl-1-pyrroline-N-oxide (DMPO) as the spin trapping agent, which is one of the most frequently used spin traps that can trap nitrogen, oxygen and carbon centered radicals^47^,^48^. The DMPO spin trap can be used to detect ^•^OH and superoxide anion radical (O_2_^•−^). Nevertheless, the DMPO adduct with O_2_^•−^ forms DMPO-OOH, and the adduct of DMPO with ^•^OH forms DMPO-OH^17^,^47^,^48^. However, DMPO-OOH can decays within 1 min to DMPO-OH. Hence, the signal initiated from DMPO-OH not only belongs to ^•^OH alone, as previously suggested^47^. We checked the ESR signals for O_2_ and N_2_ gas plasma in water using DMPO, and we observed the DMPO-OH signals in the O_2_ gas plasma and observed DMPO-OH and DMPO-H signals for the N_2_ gas plasma. As shown in Figure S3, the ESR spectrum of the O_2_ gas plasma consisted of DMPO-OH and a spin adduct derived from ^•^OH (hyperfine coupling constant, aN = 1.51; aH = 1.46 mT) while for N_2_ gas plasma we observed signals for the DMPO-OH with a hyperfine coupling constant (aN = 1.51; aH = 1.46 mT) and also the DMPO-H, a spin adduct derived from ^•^H (hyperfine coupling constant, aN = 1.68; aH = 2.24 mT), as shown in Fig. S4, that matches with that in previous studies^49^. The highest signal intensity for the DMPO-OH was detected in the O_2_ gas plasma. The amount of ^•^OH was much greater for the O_2_ gas plasma than for that determined in any of the other gas plasma samples. However, the presence of NaCl IS in O_2_ gas plasma was observed in the DMPO-OH signals, but the intensity is less than that for water signals (without IS). We did not observe any signal for the DEAP IL mixture using DMPO, and although we did not obtain DMPO-H signals for N_2_ gas plasma using IS, only DMPO-OH signals were observed with hyper fine coupling aN = 1.51; aH = 1.46 mT. Similarly for N_2_ gas plasma with DEAP IL and DMPO, we did not observe any EPR signals, which show that the intensity of the signals is affected with the presence of NaCl IS and DEAP IL in the solution.

### Structural stability analysis of Hb protein in the presence of 2% (w/v) DEAP IL and 2% (w/v) NaCl IS using molecular dynamic (MD) simulations

**Figure 5:**
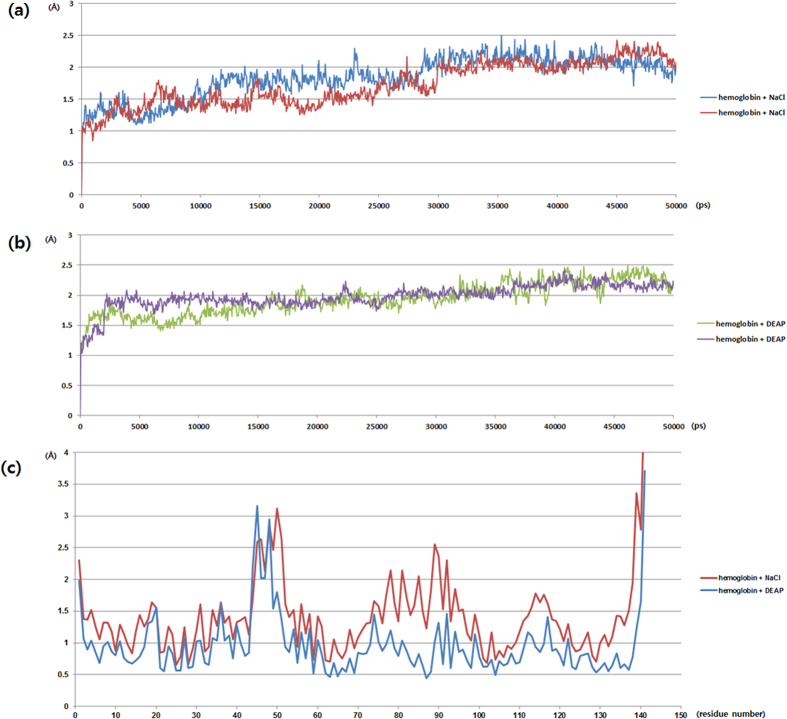
Results for the molecular dynamics simulation. (**a**) RMSD plots of NaCl soaked Hb (**b**) RMSD plots of DEAP soaked Hb and (**c**) RMSF plots of DEAP and NaCl soaked Hb.

In order to understand the molecular mechanism underlying the DEAP stabilizing Hb structure, we studied the DEAP-hemoglobin interactions through molecular dynamics (MD) simulations. For 2% (w/v) DEAP and 2% (w/v) NaCl soaked Hb, two independent simulations were performed with each lasting ~50 ns. These simulations included 10 ns of preparatory MD runs to equilibrate the components of DEAP and NaCl in the water solutions and were performed twice to validate the results of the simulation. We first calculated the root-mean-square atomic positional deviation (RMSD) values during 50 ns for the main MD simulations, as shown in Fig. 5a,b and Table 1. The DEAP and NaCl soaked Hb reached stable RMSD values after 15 ns and 30 ns, respectively, and remained at about 2 Å.

**Figure 6:**
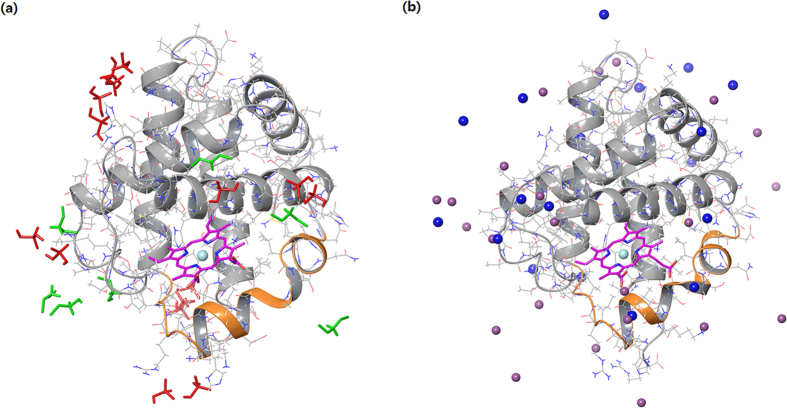
MD snapshots. (**a**) DEAP-soaked Hb, (**b**) NaCl-soaked Hb (orange ribbon: 4^th^ helix and both sides loop, purple carbon: heme, cyan sphere: Fe, blue sphere: Na. light purple sphere: Cl, green: diethylamine of DEAP, red: phosphate of DEAP).

To characterize the effects of DEAP on Hb, we calculated the root-mean-square fluctuation (RMSF) value, which describes the mean fluctuation per residue between two optimally-aligned structures. With this approach, we can measure the average atomic mobility per residue of Hb and can predict the binding region of the DEAPs. RMSF values of the two simulations shown in Fig. 5c. DEAP and NaCl bind to the surface of the Hb structure, as shown in Fig. 6a,b, respectively. When compared to NaCl-soaked Hb, DEAP-soaked Hb had lower RMSF values in most residues, which is an indication that DEAP stabilized the residue motilities of Hb. Particularly for the residues between D75-P95, the RMSF values were significantly higher (Orange ribbon of Fig. 6). The amino acid sequence of this region is DMPNALSALSDLHAHKLRVDP with seven charged residues. Since DEAP interacts with the charged residues of the protein surfaces, many DEAPs are bound to this region and significantly stabilize the region. This region for hemoglobin includes 4 helix structures and both side of the loops. His87 in this region interacts with Fe of the heme group, and therefore, one can argue that DEAP, by binding to this region of the hemoglobin binding site, keeps the hemoglobin function intact. However, during the simulations, DEAP maintained ±150 hydrogen bonds with the Hb structure and NaCl also had a similar number of hydrogen bonds. However, both cases were observed to be different (Fig. 6). The NaCl components were uniformly distributed in solutions and interacted with the Hb surface (Fig. 6b), and the DEAP components were also distributed in solutions but were clustered on the surface of Hb, as shown in Fig. 6a. The DEAP clusters made complex hydrogen bonds with hemoglobin and restrained protein motilities. Thus, DEAP stabilizes the Hb structure while NaCl does not.

### Circular dichroism and fluorescence analysis of the Hb protein structure with or without plasma in different gases and different solutes

**Figure 7:**
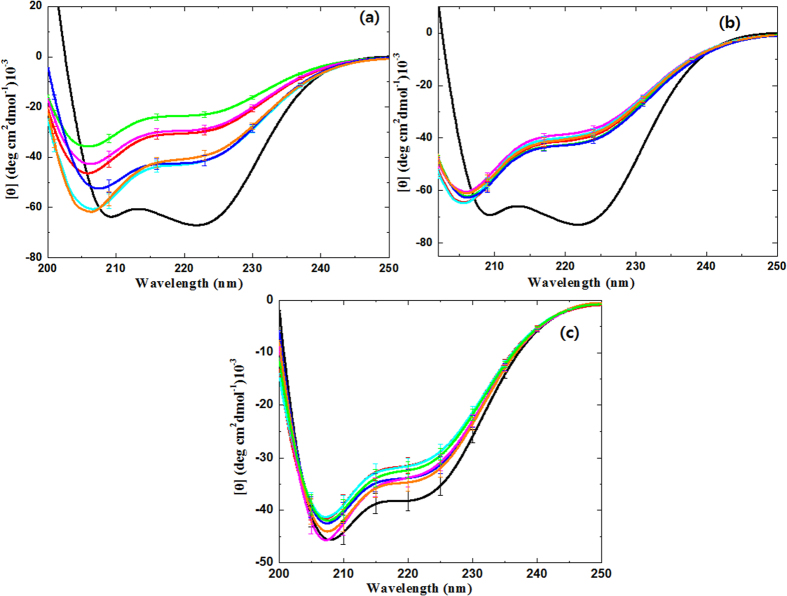
CD analysis of Hb after DBD plasma treatment in different feeding gases, such as O2 (green), Air (red), Ar (blue), He (cyan), NO (10%) +N2 (orange) and N2 (magenta) in (**a**) water; (**b**) 2% (w/v) NaCl solution and (**c**) 2% (w/v) DEAP IL solution.

In order to understand the degree of structural modification in Hb, DBD plasma was used with different feeding gases, and we checked the far-UV CD spectra of the Hb protein, as shown in Fig. 7. The far-UV CD spectra indicates the presence of a secondary structure deformation in Hb and two well-pronounced minima at ~208 and ~222 nm for Hb in water, which suggests that the polypeptide chain is mostly organized in an α-helix conformation^16^,^50^. Table S1 and Fig. 7 clearly reveal the change in the α-helix and β-sheet of the Hb protein using different feeding gases. For the control Hb, there is 69.39% of α-helix and 8.3% of β-sheet, and after treatment with O_2_ plasma, the α-helix becomes 68.81% and the β-sheet is 7.59%. For the N_2_ gas plasma, the change in the α-helix is 68.54%, and the change in the β-sheet is 7.42%. For NO (10%) + N_2_ plasma, we observed 68.97% of α-helix and 7.84% of β-sheet. This shows that there is an increase in the α-helix and a decrease in the β-sheets after treatment with different feeding gases. The smallest change in the structure is observed for Ar, He and NO (10%) + N_2_ gas plasmas, and major changes in the structure are observed for O_2_, N_2_ and Air gas plasmas. When we checked the changes in the structure with the addition of 2% (w/v) of NaCl IS and 2% (w/v) of DEAP IL, we observed different levels of structural changes. The spectra for Hb with 2% (w/v) NaCl without treatment is the same as that for the native structure, the α-helix is 69.39% and the β-sheet is 8.3% while after O_2_, N_2_, He, Ar, NO (10%) + N_2_ and Air gases, plasma the % of α-helix increases and that for the β-sheet decreases. However, the change in the structure among the different gases is very small, and this shows that the NaCl protects the protein structure irrespective of the gas that is used. On the other hand, the Hb structure changes in the presence of 2% (w/v) DEAP IL as compared to Hb in water (without treatment). However, the changes in the structure of Hb in the presence of plasma using 20% (w/v) IL are trifling.

The structure of Hb in DEAP IL is very stable, as confirmed with the MD simulations. In DEAP IL, there is 69% α-helix and 7.73% β-sheet, which shows that α-helix increases in DEAP IL. However, the change in the α-helix after O_2_, N_2_, Air, He, Ar and NO (10%) + N_2_ gases plasma is 68.96%, 68.95%, 68.98% , 68.99%, 69.00% and 69.00%, respectively. However, there is a change in the β-sheet relative to DEAP + Hb control with β-sheet changing to 7.62%, 7.69%, 7.56%, 7.57%, 7.63% and 7.67% for O_2_, N_2_, Air, He, Ar and NO (10%) + N_2_ gases plasma respectively. This shows that the α-helix of Hb did not change substantially after plasma treatment, but the β-sheet changed slightly after plasma treatment when compared with Hb in DEAP (without treatment).

Fluorescence spectroscopy has been shown to be an appropriate tool to examine the structural changes in the proteins/enzymes^16^,^22^,^35^. The quantum efficiency of tyrosine (Tyr) is much less than that for tryptophan (Trp), so the fluorescence behavior of Hb is considered according to the Trp residues. The change in the Trp emission wavelength is a result of the change in the tryptophan microenvironments that might result from the plasma action. The denaturation process or the structural deformation can be followed by changes in the maximal intensity of the fluorescence (I_max_) and the red-shift of the maximal emission wavelength (E_max_) as well as the increased polarity of the Trp residues of the protein. The maximal emission wavelength (E_max_) of Hb is ~330 nm, and this changes after plasma treatment to ~350 nm. For plasma treatment in water, there is a decrease in intensity with a red shift that shows that the Hb structure is either denatured or the Trp environment has changed significantly, as shown in Figure S5. The maximum red shift is observed for Air plasma, but quenching is the highest for O_2_ and N_2_ plasma. Quenching might be a result of the modification of the Trp group after treatment while the shift is mainly due to the change in the Trp environment and moves to a more polar environment. In the presence of NaCl, no quenching is observed after plasma treatment. An E_max_ ~330 nm is the same for NaCl as for water. After plasma treatment, we observed a red shift in the 2% (w/v) NaCl solution, which is similar to the case with water, but the shift is small for all gas plasmas. This again supports the results of the CD that structural changes of Hb in the presence of NaCl is independent of the gas used during plasma treatment. The plasma effect on the Trp group of Hb in the presence of NaCl is consists of a quite similar action for all gas plasmas. The different gas plasmas in the presence of DEAP IL have no effect on the Trp emission wavelength, except that we observed a slight red shift for the O_2_ gas plasma. This shows that the addition of IL can control the Trp environment from becoming distorted or changing due to the plasma action.

## Discussion

In the present study, we explain the role of different feeding gases in DBD plasma and their behavior in the presence of NaCl IS and DEAP IL. Furthermore, we have checked the protective action of NaCl IS and DEAP IL on the Hb protein. From the OES spectra we have observed strong peaks for N_2_ 2^nd^ positive system for the N_2_, NO (10%) + N_2_ and Air plasma. Additionally, only air plasma spectra has small emission lines from a molecular NO β, γ system between 200 and 250 nm. We also observed ^•^OH peaks for O_2_, Ar and He gas plasmas, and the intensity of the ^•^OH peak at 309.6 nm is the highest for the O_2_ gas plasma. We did not observe a clear peak for ^•^OH in the N_2_, NO (10%) + N_2_ and Air plasma, probably due to the overlap in the N_2_ 2^nd^ positive peak with ^•^OH. Moreover, we also observed an O_2_ 1^st^ positive system for the O_2_ gas plasma.

Furthermore, after treatment for 10 min with DBD plasma, the pH decreased to 3.5, and the temperature increased to 32 °C. The smaller rise in temperature shows t our system has less of a heating effect. Furthermore, we checked the evolution of ^•^OH in different gas plasmas, and from this work, we show that by providing the same energy to the DBD in different gases, we can generate different concentrations of ^•^OH in solution. We used fluorescence spectroscopy to check for the generation of ^•^OH by monitoring the conversion of TA to HTA. Our experimental data shows that the maximum HTA forms in O_2_ gas plasma, followed by N_2_, Ar, He, Air and NO (10%) +N_2_ gases plasma.

Here, we explain the probable mechanism through which OH^•^ is generated. In the O_2_ gas plasma, there are many reactions that help in the production of ^•^OH and some of the reactions are given below.


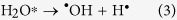



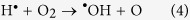



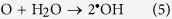



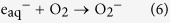



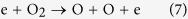



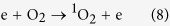



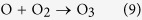



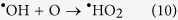



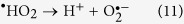



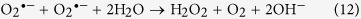



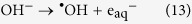



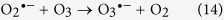



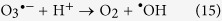



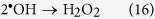



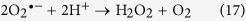



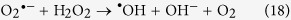


Through these reactions, we can see that the possibility of generating ^•^OH is very high for O_2_ gas plasma. On the hand, the metastable level of N_2_(A^3^∑_u_^+^) forms in N_2_ plasma, which dissociates H_2_O molecules as shown in Equation (22) below after returning back to the ground state of N_2_. In this manner, the N_2_ gas also forms a large amount of ^•^OH.


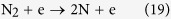



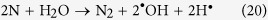



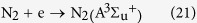



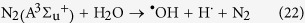


For the Ar gas plasma, Equation 3, will remain the same but we have two additional equations.


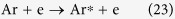



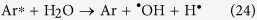


In the He gas plasma, we also have Equations 25, 26 in addition to Eq. 3.


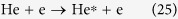



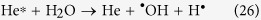


For Air gas plasma, there is the possibility to have all equations given for the O_2_ and N_2_ gas plasma.


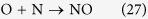



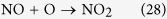



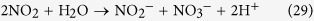



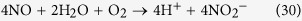


NO (10%) + N_2_ gas plasma can generate the reactions that shown in Equations 28, 29, 30 with the addition of Equation 3 to produce ^•^OH. Recently, Takamatsu *et al.* showed that nitrogen plasma produced the highest ^•^OH followed by Ar, He, O_2_ and CO_2_ plasma in PBS^17^. This trend is similar to that observed in our experiment, except for the O_2_ gas plasma.

One of the main reasons for this trend is the lifetime of the reactive species. The lifetime of the oxygen metastable state 2^1^D_2_ is 100 s, nitrogen metastable state 2^2^D_5/2_ is 6.1 × 10^4^ s, argon metastable state 4^3^P_2_ is 56 s and for helium metastable state 2^3^S_1_ is 7.9 × 10^3^ s 17. The lifetime of the metastable states can explain the presence of ^•^OH through a direct reaction, which is consistent with our data. However, there are other ways to obtain indirect equations for O_2_ gas plasma with respect to the generation of ^•^OH. As such, Equations 5, 13, 15 and 18 are mainly responsible for the increase in ^•^OH. Furthermore, we have checked the generation of ^•^OH using ESR for N_2_ and O_2_ gas plasma. We observed that DMPO-OH signals for the O_2_ gas are higher than the N_2_ signals, and additionally, we observed DMPO-H signals for N_2_ gas plasma absent in the O_2_ gas plasma. This also supports the above fluorescence data in that the O_2_ gas plasma can produce more ^•^OH.

The further generation of ^•^OH for O_2_ plasma relative to N_2_ plasma is also supported by Kohno *et al.*^49^. They show that during sonolysis of water in the presence of O_2_ gas, more ^•^OH is created than with N_2_ and Ar gas. The radicals attached with DMPO are very sensitive to the pH of the solution, and DMPO reacts with O_2_^•−^ or HO_2_^•^ to form DMPO-OOH. At the same time, DMPO reacts with ^•^OH to form DMPO-OH. However, the main idea is that the rate constant for the DMPO-OH is > 10^9^ M^−1^ s^−1^, while the rate constant for the DMPO-OOH is < 10^2^ M^−1^ s^−1^, which shows that the chance to form DMPO-OH is much higher than for DMPO-OOH. Moreover, the conversion of DMPO-OOH to DMPO-OH takes place after 1 min^47^. Hence, the intensity of the DMPO-OH signals is contributed by the O_2_^•─^, HO_2_^•^ and ^•^OH. While H^•^ generated by N_2_ plasma is in the same amount as ^•^OH. Therefore, we have observed both DMPO-H and DMPO-OH signals, and the rate constant for DMPO-H is >10^3^ M^−1^ s^−1^ 47.

Another observation is that the intensity of the ESR signals and fluorescence signals decrease with the addition of the NaCl. In our early work, we found that there is less microorganism deactivation for saline solution^33^. This shows a correlation between the presence of NaCl solution and radicals that are generated or radical interaction. When we checked the fluorescence, we observed a decrease in the intensity of HTA formation linked with the decrease in the generation of ^•^OH generation or some other reaction that decreases the effect of ^•^OH in solution. Similarly, we also observed less intense signals for EPR in the presence of the NaCl solution. The Finlayson-pitts group^51^ showed the reaction of ^•^OH with aqueous NaCl with the following equation


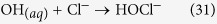



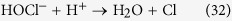



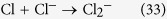



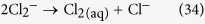



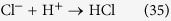


In the experiment with 2% (w/v) NaCl solution, these reactions result in the decrease in the ^•^OH reaction with TA to form HTA. Similarly, we also observed a lesser intensity in the signals for ESR in the presence of the 2% (w/v) NaCl solution. However, for the signal in the presence of the 2% (w/v) DEAP IL, the intensity of the fluorescence decreases much for all gases as compared to control and the 2% (w/v) NaCl solution. It is possible that IL interacts with the TA molecule and does not allow the ^•^OH to react with TA to form HTA. In the presence of the 2% DEAP IL solution, we did not observe ESR signals. To understand this phenomenon, we conducted semiempirical calculations and investigated the hydrogen bonding between DEAP and DMPO. As, seen from Figure S6, we can find that DEAP IL interacts with the DMPO molecule, and this interaction restricts the DMPO to interact with ^•^OH, which might be one of the main reasons for the absence of the ESR signals. The second is that DEAP IL produces interference in the ESR signals, so a further understanding on this issue will be investigated in future research.

To understand the action of NaCl IS and DEAP IL on the Hb protein, we carried out MD simulations. We found that 2% (w/v) DEAP stabilizes the Hb structure more than 2% (w/v) NaCl in the presence of plasma treatment. The CD and fluorescence results show that structural changes are different in different gases, but in the presence of NaCl, the structure for Hb did vary much in the presence of different gas plasmas. The change in the structure is also due to the change in the pH, but the change in temperature has no effect on the structure of Hb because the melting temperature of Hb is greater than the temperature change in the solution after plasma treatment^16^. In addition, the NaCl controls the changes in the Hb structure to a greater extent than the solution without NaCl. However after treatment with plasma in 2% (w/v) DEAP, the slight structural changes in Hb observed by using the CD and fluorescence spectroscopy. Hence, both DEAP and NaCl can control the Hb structure in the presence of RS generated through plasma. Nevertheless, DEAP stabilize the structure of Hb more strongly than NaCl. But we can’t predict that protective action of other ILs on proteins in the presence of plasma, because action of IL on proteins varies from IL to IL and protein to protein^52^. Similar, we can’t predict the action of the all ionic salts on proteins in the presence of plasma, because the every salt has different action with different proteins and also in the presence of plasma and salt many complex reactions can generate that result in multiple possibilities. Therefore, it need more study for understanding the action of other ionic salt and ionic liquid on various proteins, this issue will be investigated in future research.

## Experimental Section

### Materials

The Hemoglobin protein, DMPO (5, 5-dimethyl-1-pyrroline-N-oxide), sodium chloride, diethylamine and phosphoric acid (85 wt.% in H_2_O) were supplied by Aldrich Chemical Co. (USA). All chemicals and reagents were used without further purification. The synthesis of the ionic liquid is given in our previous work and supporting file^40^,^41^,^42^.

### Dielectric barrier discharge

Experiments were carried out using a scalable DBD device, as shown in Fig. 3. The device was set in a chamber equipped with a rotary vacuum pump and a gas cylinder. The gases used in the chamber were air, O_2_, NO (10%) + N_2_, N_2_, He, and Ar at atmospheric pressure. The DBD device consisted of 20 stainless rod electrodes of 1 mm in outer diameter and 60 mm in length covered with a ceramic tube of 2 mm in outer diameter. The electrodes were arranged in a parallel manner with spacing of 0.2 mm. DBD plasmas were generated between the electrodes by supplying a 10 kHz AC high voltage (Logy Electric, LHV-09K). The discharge voltage and current were measured with a high-voltage probe (Tektronix, P6015A) and a Rogowski coil (URD, CTL-28-S90-05Z-1R1), respectively. The peak-to-peak discharge voltage and current were 9.2 kV and 0.2 A, as shown in Figure S7. The corresponding discharge power density was 1.49 W/cm^2^, which was deduced from a voltage/charge Lissajous plot.

### Fluorescence spectroscopy

The fluorescence spectroscopy instrument that was used to measure the fluorescence intensity in the present investigation is similar to that depicted in our earlier work^16^,^35^. Steady-state fluorescence measurements were carried out in a Perkin Elmer LS 55 fluorescence spectrometer. The excitation wavelength was fixed at 280 nm to obtain the contribution of the Trp group. The slit width for the excitation and emissions were both set to 10 nm. The concentration for this experiment is 1 mg/ml for Hb protein.

### Circular dichroism spectroscopy

CD spectroscopic studies were performed using a J-815 spectrophotometer (Jasco, Japan) equipped with a Peltier system to control the temperature^16^,^35^. (1S)-(+)-10-camphorsulfonic acid (Aldrich, Milwaukee, WI) was used for the CD calibrations, exhibiting a molar extinction coefficient of 34.5 M/cm at 285 nm, and molar ellipticity (θ) of 2.36 M/cm at 295 nm. The samples were pre-equilibrated at the desired temperature for 15 min, and the scan speed was fixed for adaptive sampling (error F 0.01) with a response time of 1 s with 1 nm bandwidth. The secondary Hb structures were monitored using a 1.0 cm path length cuvette. The concentration for the secondary Hb structure was 0.1 mg/ml, with each spectrum being the average of six spectra. Each sample spectrum was obtained by subtracting the appropriate blank media without Hb from the experimental protein spectrum. The percentages of the secondary structures were then calculated using the K2D3 online software.

### EPR analysis

EPR spectroscopy is a measurement technique that is used to detect unpaired electrons. After plasma treatment, the DMPO aqueous solution was transferred to a quartz cell for EPR spectrometry, and then the EPR spectrum was immediately recorded on an X band EPR spectrometer (JES-FA-100, JEOL, Tokyo, Japan). The measurement conditions for EPR were as follows: field sweep, 330.50–340.50 mT; field modulation frequency, 100 kHz; field modulation width, 0.1 mT; amplitude, 20; sweep time, 2 min; time constant, 0.03 sec; microwave frequency, 9.421 GHz: microwave power, 4 mW. The EPR spectrum of manganese (Mn^2+^), which was equipped in the EPR cavity, was used as the internal standard. 2 ml of treated volume were used for each experiment, and each treatment was performed three times. The signal decay was determined only as an example for a 74 h a spectrum. The procedure for the experiments with DMPO were as follows. Each time before a measurement was performed, a fresh DMPO solution (100 mM) was prepared. From this solution, a sample of up to 500 μl was taken and was placed into the borosilicate glass sample tube.

### Ion analysis, pH and temperature measurement

After the plasma was exposed for 10 min in water, the ion analysis, pH and temperature of the water were measured using a Thermo Scientific™ Dionex™ ion chromatography (IC) system, a pH meter (Eutech Instruments, Singapore) and an Infrared (IR) camera (Fluke Ti100 Series Thermal Imaging Cameras, UK). All measurements were carried out in triplicate.

### Molecular Dynamics Simulation

The structure file for human hemoglobin (PDB ID: 1A3N) was obtained from the protein data bank (PDB) website (http://www.rcsb.org). The structure was prepared with the Protein Preparation Wizard^53^ of the Schrödinger suite for the MD simulations. During this preparation, only one hemoglobin subunit was left and all water and het molecules were eliminated. The missing hydrogens were added to obtain the proper pKa values^54^ and were minimized using IMPACT^55^. All molecular dynamics (MD) simulations and analyses were performed using the DESMOND simulation package^56^ and Maestro graphical interface^57^. The simulation systems were built using the Desmond system builder. In this step, the system was solvated with a TIP3P model water, and DEAP and NaCl molecules were added to the system with concentrations in an experimental setting. Approximately 6800 water molecules were included in a periodic box with a size of 6.4 × 4 × 6 nm^3^. The DEAP charges were fitted to the ESP charges which were then calculated using Jaguar^58^ at B3LYP/6-31G**. The total charges of the system were neutralized by adding the ions. An OPLS2005 all atom force field was used for all simulations. A Short-range cutoff distance of 9 Å was applied for the Coulombic interactions with the inclusion of the particle mesh Ewald (PME) summation for long-range interactions. The temperature and the pressure were maintained at a reference temperature of 298 K with a pressure of 1atm by applying the Nose-Hoover thermostat and the Martyna-Tobias-Klein barostat in the NPT ensemble. Before the main simulations, we performed a series of short minimizations and 10 ns equilibrium MD runs.

### Hydrogen bonding analysis through Simulation program

The structures of IL and DMPO were optimized based on molecular mechanics and semi-empirical calculations using the HyperChem 7 molecular visualization and simulation program. The details are provided in our early work^34^.

### Sample Preparation

The protein stability was analysed by incubating 2 ml screw-capped vials in deionized water, at 25 °C for 4 h to attain complete equilibrium. The samples were treated at 6 mm distances from the plasma for 10 min and were then incubated for 4 h at room temperature. Three samples were treated for each condition to minimize the error.

### Statistical analysis

All of the values are represented by the mean ± S.D of the indicated number of replicates, and the statistical analyses of the data were performed using Student’s t-test to establish significance between the data points with significant differences based on P < 0.05.

## Additional Information

**How to cite this article**: Attri, P. *et al.* Influence of ionic liquid and ionic salt on protein against the reactive species generated using dielectric barrier discharge plasma. *Sci. Rep.* **5**, 17781; doi: 10.1038/srep17781 (2015).

## Supplementary Material

Supplementary Information

## Figures and Tables

**Table 1:** Average and standard deviation of root-mean-square atomic positional deviation (RMSD).

	Hemoglobin protein +2% (w/v) NaCl	Hemoglobin protein +2% (w/v) DEAP
Avg	1.838	1.934
	1.724	1.983
Stdev	0.318	0.267
	0.341	0.202
