# Supplementary material for: Influence of ionic liquid and ionic salt on protein against the reactive species generated using dielectric barrier discharge plasma
Source: Sci Rep. 2015 Dec 10;5:17781. doi: 10.1038/srep17781 (PMC4674715; doi:10.1038/srep17781)
Supplement: Supplementary Information [file srep17781-s1.pdf]

Supporting Information

Influence of ionic liquid and ionic salt on protein against the reactive species generated using dielectric barrier discharge plasma

Pankaj Attri[1,2], Thapanut Sarinont[2], Minsup Kim[3], Takaaki Amano[2], Kazunori Koga[2]*, Art E. Cho[3]*, Eun Ha Choi[1]*, Masaharu Shiratani[2]*

[1]Plasma Bioscience Research Center / Department of Electrical and Biological Physics, Kwangwoon University, Seoul, Korea.
[2] Graduate School of Information Science and Electrical Engineering, Kyushu University Fukuoka, Japan
[3]Department of Bioinformatics, Korea University, Sejong, Korea

Synthesis of Diethylammonium dihydrogen phosphate (DEAP)

The synthesis of ionic liquids was carried out in a 250 ml round-bottomed flask, which was immersed in a water-bath and fitted with a reflux condenser. Phosphoric acid (1 mol) was dropped into the diethylamine (1 mol) at 70 ^{0}C for 1 h. The reaction mixture was heated at 80 ^{0}C with stirring for 2 h to ensure that the reaction had proceeded to completion. The reaction mixture was then dried at 80 ^{0}C until the weight of the residue was constant.

Figure Captions

Figure S1: Physical and chemical parameters changed after the plasma treatment in different gases plasma such as (a) pH; (b) temperature; (c) NO_2^- and (d) NO_3^-.

Figure S2: Fluorescence analysis of OH radicals using TA solution in (a) water; (b) 2 % (w/v) NaCl solution and (c) 2 % (w/v) DEAP IL solution after treatment with DBD plasma using O_2 feeding gas.

Figure S3: ESR spectra of OH radicals in the presence of O_2 plasma for 10 min treatment with or without presence of 2 % NaCl.

Figure S4: ESR spectra of OH and H radicals in the presence of N_2 plasma for 10 min treatment with or without presence of 2 % NaCl.

Figure S5: Fluorescence analysis of Hb after treatment with different gases plasma (a) Hb treatment in water; (b) Hb treatment in the presence of 2% NaCl solution and (c) Hb treatment in the presence of 2% DEAP IL solution after DBD plasma treatment with different feeding gases, such as Air (red), Ar (blue), He (cyan), NO (10%) + N_2 (orange) and N_2 (magenta).

Figure S6: Schematic depiction of the Hydrogen bonding of DEAP and DMPO.

Figure S7: Schematic depiction of (a) dimension of the DBD used in current work and (b) Current & voltage graph of DBD.

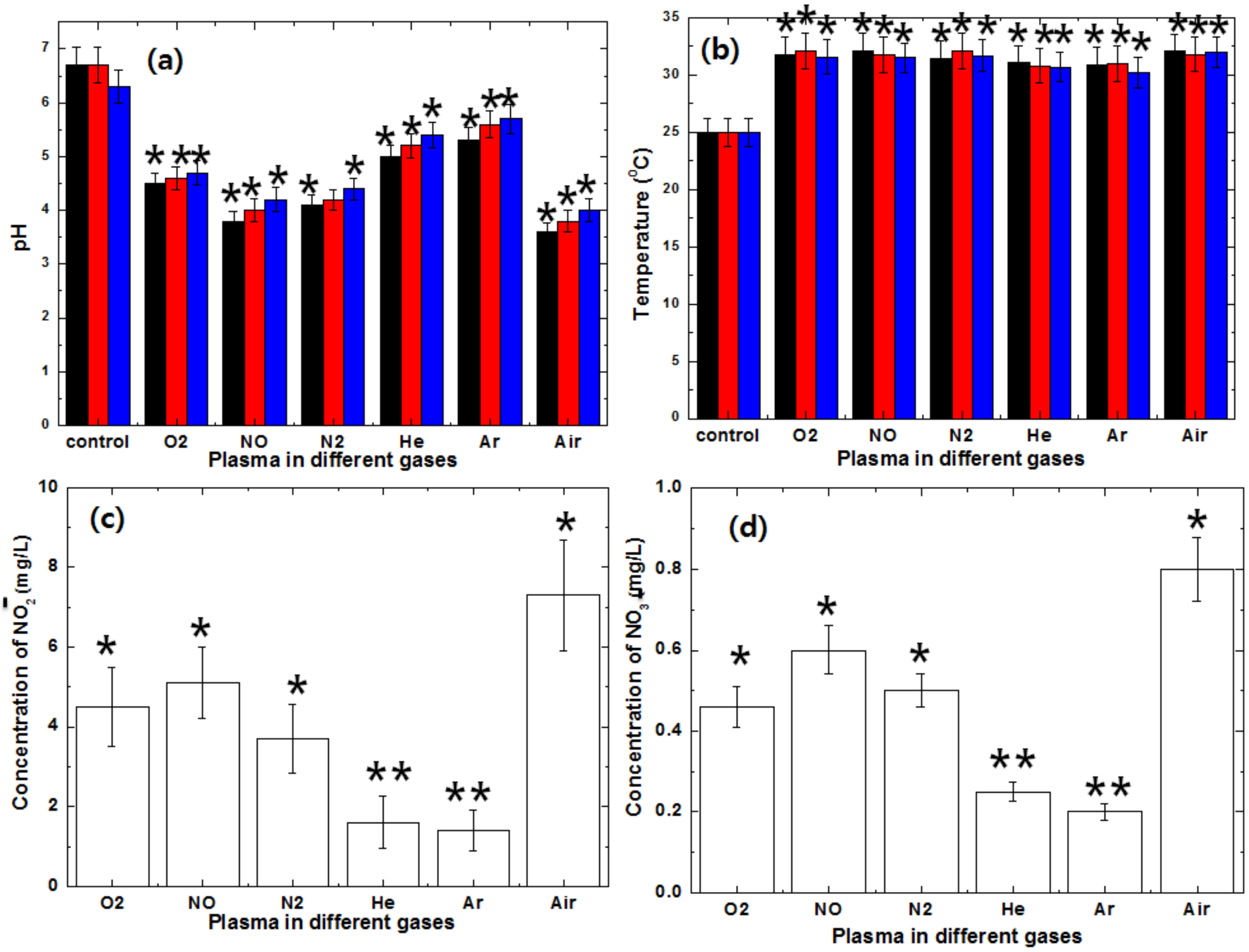

Figure S1

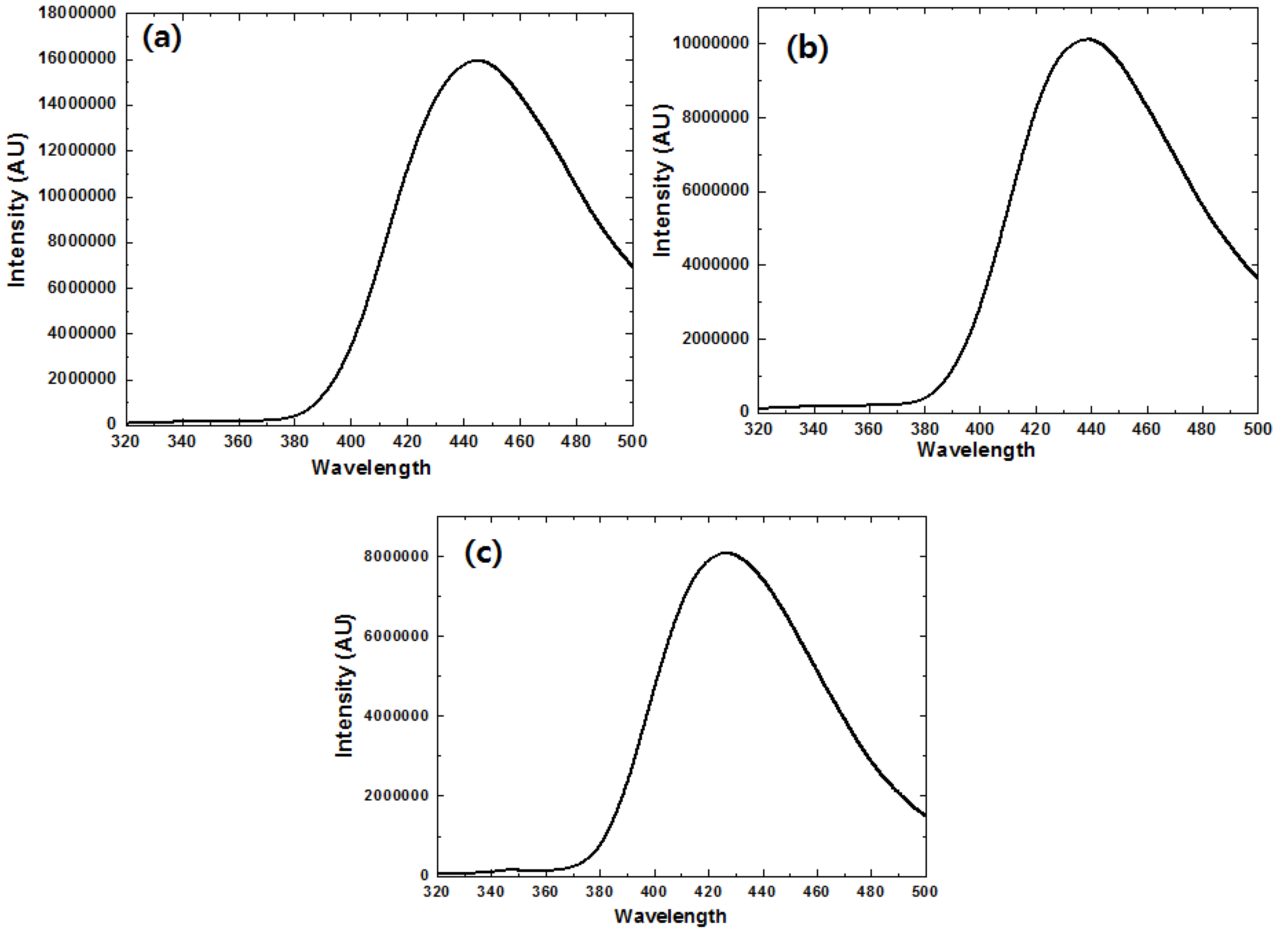
(a)
Intensity (AU)
Wavelength
0
2000000
4000000
6000000
8000000
10000000
12000000
14000000
16000000
18000000
320 340 360 380 400 420 440 460 480 500
(b)
Intensity (AU)
Wavelength
0
2000000
4000000
6000000
8000000
10000000
320 340 360 380 400 420 440 460 480 500
(c)
Intensity (AU)
Wavelength
0
2000000
4000000
6000000
8000000
320 340 360 380 400 420 440 460 480 500

Figure S2

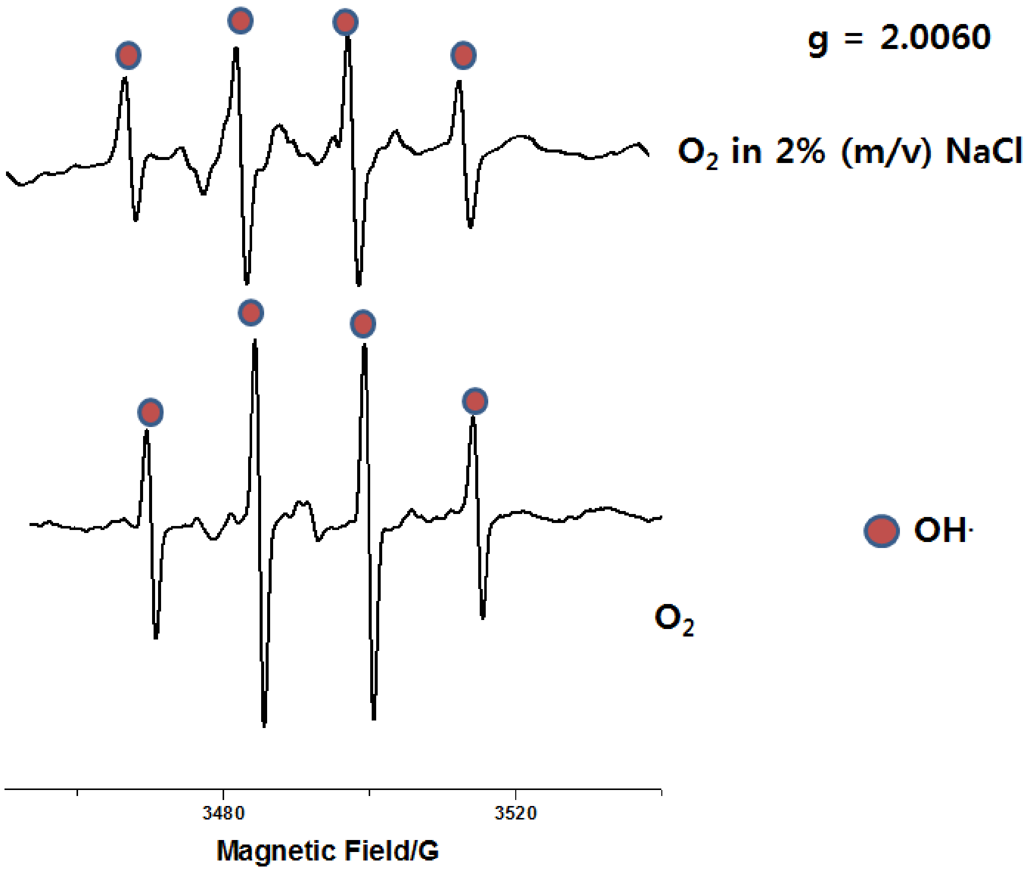

Figure S3

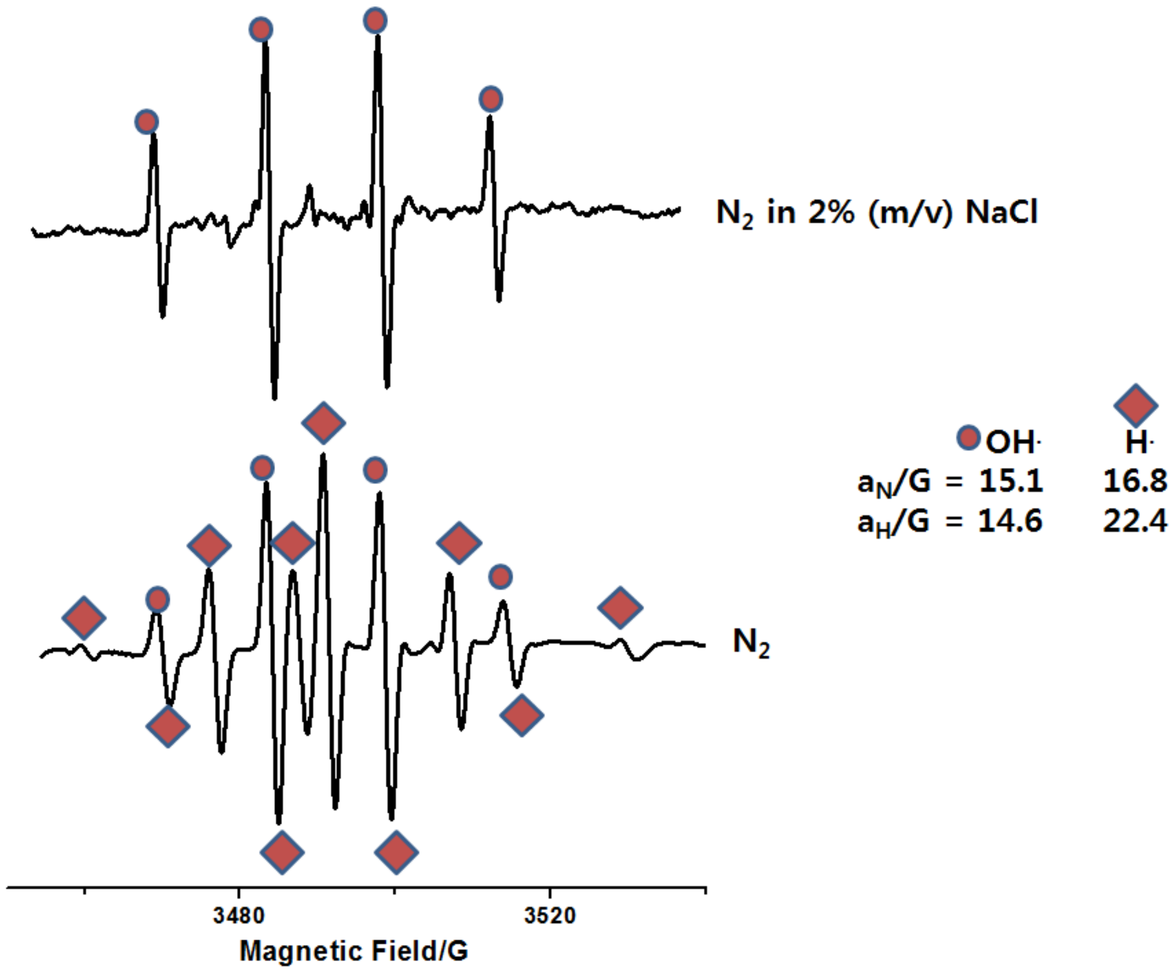
N2 in 2% (m/v) NaCl
OH·
H·
aN/G = 15.1
16.8
aH/G = 14.6
22.4
N2
3480
3520
Magnetic Field/G

Figure S4

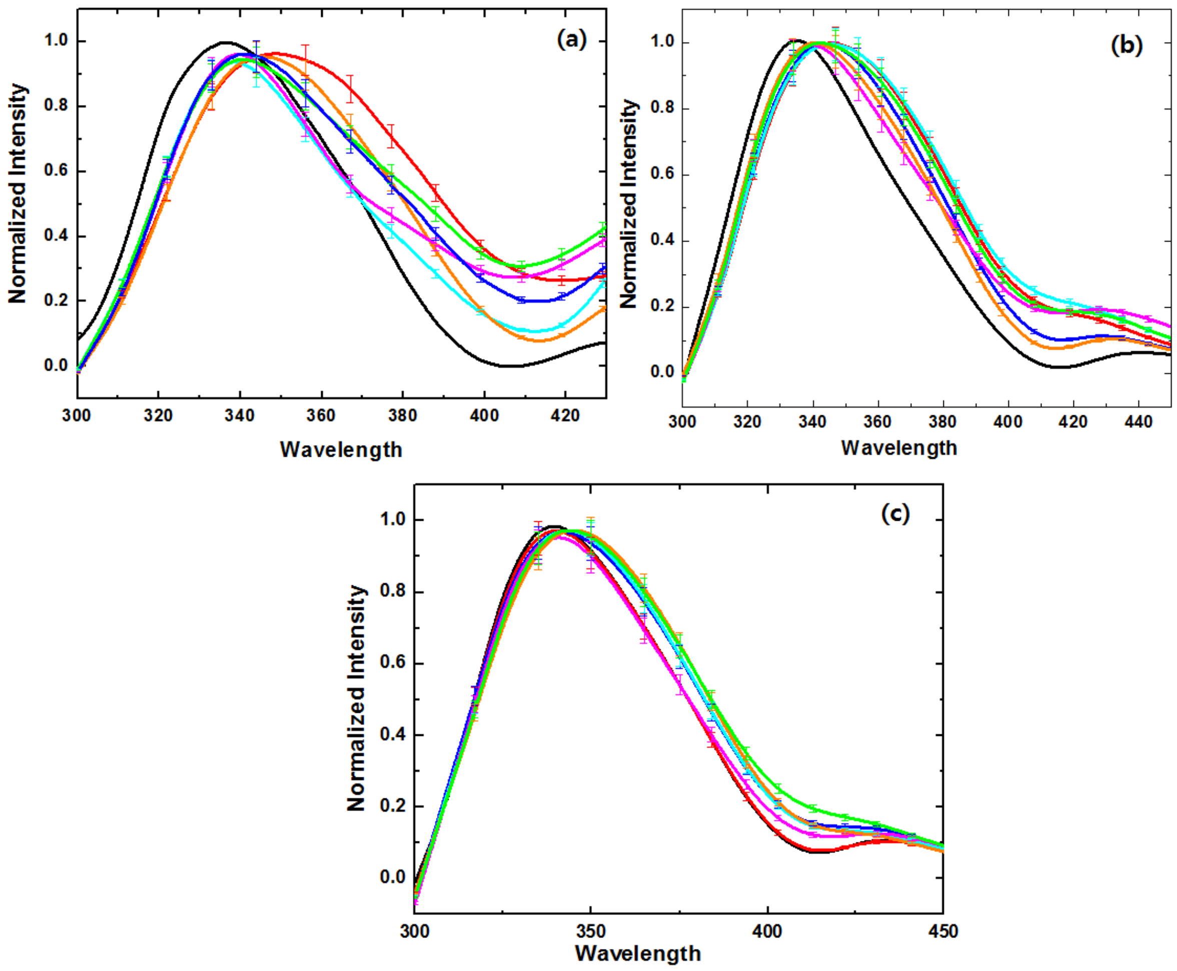
(a)
Normalized Intensity
Wavelength
1.0
0.8
0.6
0.4
0.2
0.0
300
320
340
360
380
400
420
(b)
Normalized Intensity
Wavelength
1.0
0.8
0.6
0.4
0.2
0.0
300
320
340
360
380
400
420
440
(c)
Normalized Intensity
Wavelength
1.0
0.8
0.6
0.4
0.2
0.0
300
350
400
450

Figure S5

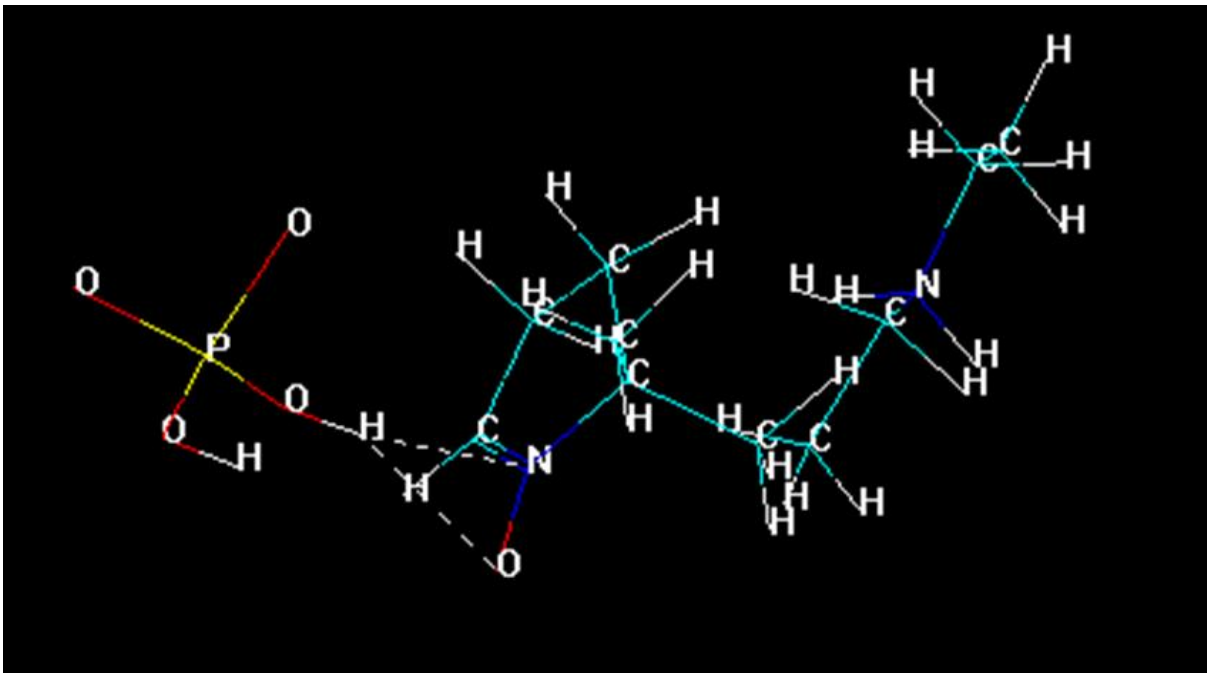

Figure S6

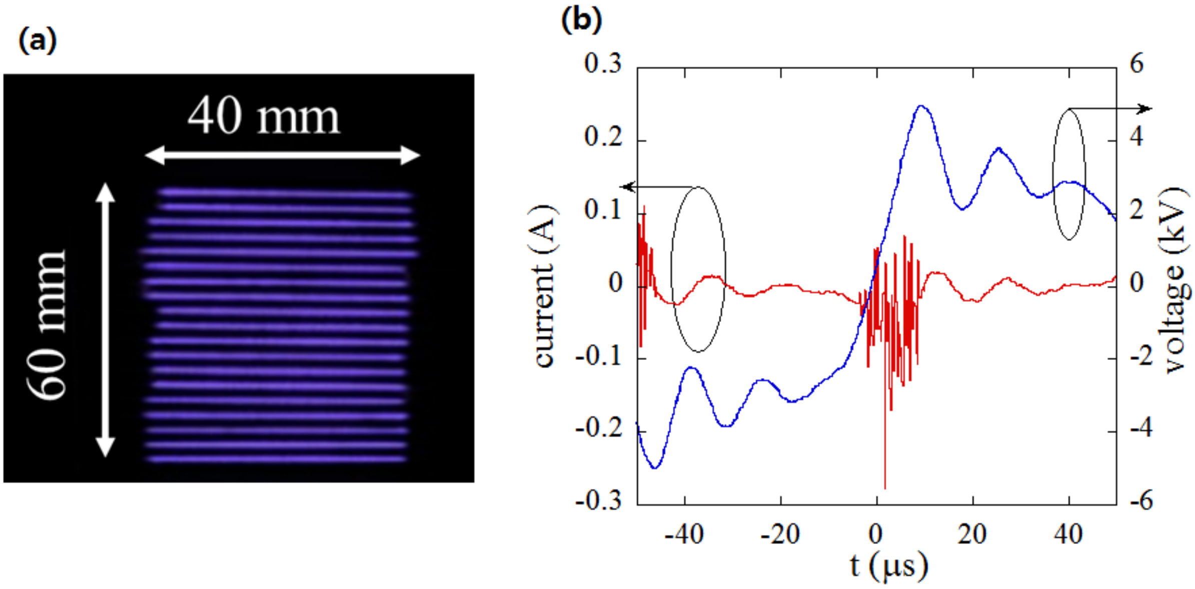

Figure S7

Table S1. Secondary structure composition of Hb, determined from Far UV CD spectra in different solvent condition and with different feeding gases at 20 ^{0}C determined by K2D3.

Sample	α-sheet (%)	β-sheet (%)
Hb	69.39	8.3
Hb + Air	68.79	7.6
Hb + N_2	68.54	7.42
Hb + NO (10%) + N_2	68.97	7.84
Hb + O_2	68.81	7.59
Hb + He	68.89	7.82
Hb + Ar	68.92	7.8
Hb + Air + 2% NaCl	69.03	7.52
Hb + N_2+ 2% NaCl	68.95	7.49
Hb + NO + 2% NaCl	68.79	7.57
Hb + O_2+ 2% NaCl	69.10	7.54
Hb + He+ 2% NaCl	68.95	7.52
Hb + Ar+ 2% NaCl	69.05	7.56
Hb + 2% DEAP	69.00	7.73
Hb + Air + 2% DEAP	68.98	7.56
Hb + N_2+ 2% DEAP	68.95	7.69
Hb + NO (10%) + N_2 + 2% DEAP	69.00	7.67
Hb + O_2+ 2% DEAP	68.96	7.362
Hb + He+ 2% DEAP	68.99	7.57
Hb + Ar+ 2% DEAP	69.00	7.63
